# Flower Production, Headspace Volatiles, Pollen Nutrients, and Florivory in *Tanacetum vulgare* Chemotypes

**DOI:** 10.3389/fpls.2020.611877

**Published:** 2021-01-20

**Authors:** Elisabeth J. Eilers, Sandra Kleine, Silvia Eckert, Simon Waldherr, Caroline Müller

**Affiliations:** ^1^Chemical Ecology, Bielefeld University, Bielefeld, Germany; ^2^Biodiversity Research/Systematic Botany, University of Potsdam, Potsdam, Germany

**Keywords:** terpenoids, gas chromatography-mass spectrometry (GC-MS), Asteraceae, protein:lipid-ratio, insect behavior, Phalacridae, chemodiversity

## Abstract

Floral volatiles and reward traits are major drivers for the behavior of mutualistic as well as antagonistic flower visitors, i.e., pollinators and florivores. These floral traits differ tremendously between species, but intraspecific differences and their consequences on organism interactions remain largely unknown. Floral volatile compounds, such as terpenoids, function as cues to advertise rewards to pollinators, but should at the same time also repel florivores. The reward composition, e.g., protein and lipid contents in pollen, differs between individuals of distinct plant families. Whether the nutritional value of rewards within the same plant species is linked to their chemotypes, which differ in their pattern of specialized metabolites, has yet not been investigated. In the present study, we compared *Tanacetum vulgare* plants of five terpenoid chemotypes with regard to flower production, floral headspace volatiles, pollen macronutrient and terpenoid content, and floral attractiveness to florivorous beetles. Our analyses revealed remarkable differences between the chemotypes in the amount and diameter of flower heads, duration of bloom period, and pollen nutritional quality. The floral headspace composition of pollen-producing mature flowers, but not of premature flowers, was correlated to that of pollen and leaves in the same plant individual. For two chemotypes, florivorous beetles discriminated between the scent of mature and premature flower heads and preferred the latter. In semi-field experiments, the abundance of florivorous beetles and flower tissue miners differed between *T. vulgare* chemotypes. Moreover, the scent environment affected the choice and beetles were more abundant in homogenous plots composed of one single chemotype than in plots with different neighboring chemotypes. In conclusion, flower production, floral metabolic composition and pollen quality varied to a remarkable extend within the species *T. vulgare*, and the attractiveness of floral scent differed also intra-individually with floral ontogeny. We found evidence for a trade-off between pollen lipid content and pollen amount on a per-plant-level. Our study highlights that chemotypes which are more susceptible to florivory are less attacked when they grow in the neighborhood of other chemotypes and thus gain a benefit from high overall chemodiversity.

## Introduction

Outcrossing plants attract pollinators by various traits such as color, odor and nutritious reward traits, i.e., nectar and pollen. However, these rewards are likewise explored by florivores that consume pollen and floral tissues prior to seed maturity (McCall and Irwin, 2006), which may lead to contrasting selection pressures (Theis and Lerdau, 2003; Schiestl, 2015) and thus potentially also high variation. Indeed, the extent to which the composition and concentrations of primary metabolites such as amino acids and sugars within reward traits and specialized metabolites (i.e., compounds that are often restricted to certain plant taxa, formally known as secondary metabolites) such as headspace volatile compounds (Knudsen et al., 2006) and pigments within floral tissues (Harborne and Smith, 1972) differ across plant species is remarkable (Borghi et al., 2017). For example, more than 1700 volatile chemicals have been identified from floral headspace samples of different plant species (Knudsen et al., 2006), with terpenoids and benzenoids occurring most frequently (Knudsen et al., 2006; Farré-Armengol et al., 2015, 2020). The species-specific floral scent bouquet is in the majority of cases an attractant, but not always an honest signal for the presence of reward traits such as nectar and/or pollen (Knauer and Schiestl, 2015) and enables flower visitors to identify their host flowers. While variation in metabolic composition between plant species is well described, little is known about how this chemicals vary among individuals within species and affect the individual floral scent, reward trait composition and thus also the host finding ability of flower visitors.

Plants are bound to attract pollinators while repelling herbivores by adapting visual, olfactory cues and reward trait composition to avoid excess pollen removal by pollen-feeders (Hargreaves et al., 2009; Junker, 2016). One commonly described adaptation to avoid pollen robbery is the concurrent and fine-tuned provision of attractive combined with defensive components (Irwin et al., 2014), such as specific repellents against generalist florivores (Kessler et al., 2013). The latter study found that *Petunia* flowers emit a complex blend containing, amongst other compounds, isoeugenol and benzyl benzoate which deter florivores and methyl benzoate which attracts florivores and pollinators. In general, (mono-)terpenoids are evolutionarily regarded as antagonist-defensive compounds (Junker and Blüthgen, 2010; Junker et al., 2010; Schiestl, 2010). However, particularly terpenoids are highly diverse in function and the same compound may attract one animal but repel another, depending on context and released amount by the flowers (Raguso, 2016). Additionally, even within an individual plant, floral scent components show spatial and temporal variation. The scent bouquet may differ between flower parts (MacTavish et al., 2000; Dötterl and Jürgens, 2005; Flamini et al., 2007), may change after successful pollination (Tollsten and Bergström, 1989; Negre et al., 2003), after herbivory (Hoffmeister et al., 2015; Hoffmeister and Junker, 2017), with the time of day (Theis et al., 2007; Martel et al., 2019), or with floral ontogeny (reviewed in Piechulla and Effmert, 2010).

Apart from floral scent emission, pollen production is essential for sexual reproduction in both self-incompatible and animal-pollinated plants but also resource demanding, as flower visitors often remove huge amounts (Schlindwein et al., 2005; Müller et al., 2006). Chemically, pollen contains higher levels of proteins, lipids, vitamins, and minerals than nectar and various flower visitors are obligate pollen feeders (Nicolson, 2011). The plant’s phylogenetic background and pollinator dependency shape the composition of pollen proteins and lipids, which differ greatly between plant families (Vaudo et al., 2020). Pollinators can adapt their foraging strategies to the macronutrient composition of pollen, namely the protein:lipid-ratio (Vaudo et al., 2016). In addition, pollen of various plant species also contains toxins (Rivest and Forrest, 2020), but the extent of intraspecific variation in the composition of pollen macronutrients and (toxic) specialized metabolites remains largely unknown.

The chemical ecology of Asteraceae pollen is particularly interesting. Species within Asteraceae produce comparatively high amounts of pollen per floral unit (Hicks et al., 2016) and pollinator specialization to Asteraceae pollen occurs in multiple bee lineages (Müller and Kuhlmann, 2008; Praz et al., 2008a; Cane, 2017). Paradoxically, pollen of many Asteraceae is poor in proteins (Nicolson and Human, 2013; Vaudo et al., 2020), harmful to bees (Schmidt et al., 1987; Tasei and Aupinel, 2008; Vanderplanck et al., 2020), and even Asteraceae-specialists may suffer from feeding on Asteraceae pollen from a single plant species (here: *Tanacetum vulgare;* Praz et al., 2008b).

In the present study, we investigated the intraspecific variation in flower traits of *Tanacetum vulgare* (common tansy, Asteraceae) and effects on florivores. This plant species contains an exceptional intraspecific chemodiversity (i.e., expression of various different monoterpenoid-chemotypes) in the leaves (Wolf et al., 2012a; Kleine and Müller, 2013; Clancy et al., 2018) and flowers (Gabel et al., 1992). In natural populations, mono-chemotypes, i.e., with one dominant terpenoid of more than 50% relative abundance of the overall terpenoid content, co-occur with plants of mixed chemotypes (Kleine and Müller, 2011). *Tanacetum vulgare* is self-incompatible and highly dependent on pollinators for seed production, which are rewarded with pollen (Lokki et al., 1973). Florivorous beetles of the genus Phalacridae (shining flower beetles, i.e., *Olibrus* spp.) commonly occur on *T. vulgare* inflorescences (Schmitz, 1998, own personal observation). Adult *Olibrus* beetles feed on pollen and oviposit in the flower heads, and the larvae consume unripe seeds (Müller-Schärer and Brown, 1995). Foliar herbivores such as beetles (Wolf et al., 2012b) or aphids (Völkl et al., 1999; Kleine and Müller, 2011; Benedek et al., 2015) prefer certain chemotypes and plant parts (Jakobs and Müller, 2018, 2019; Jakobs et al., 2019). However, the effects of intraspecific chemodiversity in *T. vulgare* on flower-visiting insects have yet not been elucidated.

We hypothesized that *T. vulgare* plants express chemotype-specific patterns of volatile organic compounds in the floral and pollen headspace, which differ in compound diversity, i.e., chemodiversity. Individuals with a high floral headspace chemodiversity were expected to produce lower pollen amounts, due to higher enzyme costs (Gershenzon, 1994). Furthermore, we expected the contents of pollen macronutrients to differ between chemotypes and trade-off with the blooming duration and the amount of pollen per plant individual, due to resource limitations. Emission of attractive cues for (pollinating) insects is only relevant for plants during anthesis. In contrast, premature flowers should comprise a non-attractive or even deterrent scent bouquet to fight off florivores, which are present at all floral developmental stages. We therefore hypothesized that within-plant composition of volatile compounds changes with floral ontogeny and thus that florivorous beetles prefer volatile cues of mature, pollen-producing flower heads to those of unripe flower heads. These hypotheses were tested in laboratory experiments. In addition, we expected that *T. vulgare* chemotypes vary in susceptibility to florivores under semi-field conditions when grown in homogenous stands of the same chemotype. These differences in susceptibility should decrease in heterogenous stands comprising plants of different chemotypes, because of overlapping odor plumes, and thus lower concentrations and potential masking of single repellent or deterrent compounds and/or a higher overall chemodiversity (Riffell et al., 2014; Cai et al., 2017).

## Materials and Methods

### Plant Cultivation, Chemotype Determination and Selection

For semi-field experiments, *T. vulgare* seeds were collected in November 2011 close to the experimental site in Bielefeld Ummeln (Germany, 51°58′58.52′N, 8°27′12.27′E, elevation 104 m; see section “Semi-Field Experiment: Florivore Abundance and Flower Head Miner Counts”). For laboratory and greenhouse experiments, seeds were collected in January 2019 at the same site and three additional sites nearby (51°58′51.8′N, 8°27′40.0′E; 51°58′59.3′N, 8°28′13.8′E; 51°58′42.2′N 8°28′35.5′E; elevation 98–105 m). The area of each collection site was <1 km^2^ to ensure comparable local climate conditions and the distance between plants was maximized to reduce genetic, and thus chemotypic, similarity of seeds (see Lokki et al., 1973, for a chemotypic analysis of chemotype crossings). In both years, seeds of at least 10 mother plants per site were germinated 1–6 weeks after seed collection on glass beads and seedlings were transferred to pots filled with a 2:1 mixture of sterilized potting soil and river sand. Four weeks after germination, the offspring (F1) plants were in each case transferred to larger pots with the same substrate as in Jakobs et al. (2019).

Chemotypes were defined as mono-chemotypes based on the most abundant terpenoids and high similarity in satellite compounds, i.e., terpenoids present in lower abundance. Chemotype determination of all plants was conducted by the same procedure: Leaflets of the second oldest leaf of a 6–8 weeks old plant were frozen in liquid nitrogen, lyophilized and extracted by homogenization in 1 mL *n*-heptane (99% HPLC grade, Roth, D) containing 50 ng μL^–1^ 1-bromodecane (97% GC-MS grade, Sigma Aldrich, D) as internal standard with a stainless steel ball (Ø 5 mm) for 30 s at 30 Hz in a mixer mill (MM 301, Retsch, D), followed by ultrasonic bathing at 20°C for 15 min (RK 100 H, Bandelin Sonorex, D) and centrifugation at 13200 rpm, 20°C for 5 min (modified after Kleine and Müller, 2011; Wolf et al., 2012a; Jakobs and Müller, 2018). The supernatants were analyzed using gas chromatography coupled with mass spectrometry (GC-MS; see section “Measurements by GC-MS”). For the semi-field experiments in 2012, four mono-chemotypes of high abundance in the field were chosen: β-thujone, *(E)-*carvyl acetate, (Z)-chrysanthenyl acetate and camphor. For the laboratory and greenhouse experiments in 2019 and 2020, the two most common mono-chemotypes (β-thujone and artemisia ketone), and the three most common mixed-chemotypes were chosen, which contained ≥ 50% of all terpenoids of α- and β-thujone, artemisia ketone and artemisia acetate, and myroxide, artemisia acetate and santolina triene. In general, vegetative propagated plants of *T. vulgare* maintain a stable leaf terpenoid composition (Clancy et al., 2018, personal observations). Similarly, the monoterpenoids with highest compound abundance in leaf extracts had also the highest abundance in flower head extracts, so that the leaf chemotypes corresponded to flower chemotypes. This was verified for a random sample of 20 plants from the greenhouse, i.e., four of each chemotype. All plants were grown in the greenhouse at 21°C and 16 h:8 h, light:dark, fertilized weekly (modified solution after Arnon and Hoagland, 1940) and transferred after chemotype determination either to a greenhouse chamber with equal conditions or to the field site.

### Measurements of Flower and Pollen Traits

The greenhouse-grown plants flowered over a period of 4 months with a blooming period between 2 and 4 weeks. We defined flower heads, i.e., capitula, as premature once the buds were open but pollen was not yet produced and as mature when pollen was visible but flowers were not senescent (Dupont et al., 2018). Thrice a week, premature and mature flower heads were counted for each plant (*N* = 150, 30 plants per chemotype). Additionally, the total number of flower heads, the diameter of flower heads (mm), the onset and duration of the blooming period (day number, where day 0 was defined as the day of the first observed flowering) as well as the amount of pollen per day (mg) and the total amount of pollen (mg) were determined over the whole blooming period for all flowering plants. Per chemotype, 9–16 plants were sampled from the beginning of flowering to complete withering. Pollen of remaining late-flowering plants as well as pollen of plants that showed a second blooming period were not considered for analyses but used for the florivore choice bioassay (see section “Florivorous Beetles and Four-Field Olfactometer Choice Assay”). As central and lateral flower heads within one inflorescence may differ, e.g., in the probability of setting fruit (Guitián and Navarro, 1996), pollen was pooled from all flower heads with dehisced anthers per plant. For pollen collection, the flower heads were gently shaken over a piece of weighing paper, from which the pollen was transferred to a centrifuge tube and kept on ice. The pollen was weighted within 1 h and stored at −80°C for 2–6 weeks.

Protocols after Roulston et al. (2000); Laurentin and Edwards (2003), and Vaudo et al. (2016) were modified to extract and quantify protein, carbohydrate and lipid contents (i.e., macronutrients) in microtiter plate–photometer assays. Pollen samples were dried for 24 h at 36°C and weighted again to determine the water content. Proteins were extracted from 1 mg dried pollen in 500 μL cold 0.1 mol L^–1^ NaOH by homogenizing the pollen, followed by 15 min ultrasonic bath incubation and centrifugation for 5 min at 13400 rpm. Supernatants were 1:5 diluted in 0.1 mol/L NaOH, and 30 μL were combined with 200 μL Coomassie Brilliant Blue G-250 (AppliChem GmbH, D) in triplicates for each sample in a microtiter plate. Absorbance was measured at 595 nm in a plate reader (Thermo Scientific, United States), using a bovine serum albumin (Roth, D) concentration series as standard (Bradford, 1976). We extracted carbohydrates and lipids together from 2 mg dried pollen samples by homogenization in 200 μL 2% Na_2_SO_4_. After adding 1 mL of chloroform:methanol (1:1 v:v), samples were placed for 15 min in an ultrasonic bath and then centrifuged for 5 min at 13,400 rpm. Water (375 μL) was added to the supernatants (each approximately 400 μL) and samples were vortexed and centrifuged again to obtain two phases, which were separated. We doubly concentrated the upper methanol/water phase (approximately 400 μL) containing the carbohydrates under vacuum at 60°C. As carbohydrate standard, a glucose (Roth, D) concentration series in water was used. Of each carbohydrate sample and glucose standard, triplicates of 40 μL were each combined with 100 μL 2 g L^–1^ anthrone in 98% sulfuric acid in microtiter plates (Immulon 4, Dynatech Laboratories Ltd., United Kingdom), foil-sealed, shaken for 20 s in the photometer and incubated for 3 min at 92°C. The samples were left for 5 min at room temperature, incubated for 15 min at 45°C and absorbance was measured at 595 nm. As standard for lipids, a linseed oil (Naturwert Bio, D) concentration series in chloroform was used. Lipid samples (chloroform phase, approximately 165 μL) and equal volumes of linseed oil standards were dried under vacuum at 60°C and each resolved in 200 μL 98% sulfuric acid by vortexing for 10 s. Of each sample and standard, 50 μL triplicates were applied on microtiter plates, sealed, and incubated for 12 min at 92°C. After a water bath for 5 min at 18°C, plates were unsealed and 100 μL of 400 μg mL^–1^ vanillin in 34% phosphoric acid was added to each well. Then, samples were left for 10 min at room temperature and the absorbance was measured at 570 nm. For pollen terpenoid analyses, 15 ± 5 mg fresh pollen per plant was solved in 100 μL of *n*-hexane containing 50 ng μL^–1^ 1-bromodecane as internal standard and shaken at 350 rpm for 60 min at 20°C (modified after Wiese et al., 2018), then centrifuged at 13200 rpm, 20°C for 5 min and the supernatant was transferred into glass vials for analysis of compounds with GC-MS (see “Measurements by GC-MS”).

### Floral Headspace Volatile Collection

Floral headspace volatile organic compounds (VOC) were trapped on absorbent polydimethylsiloxane (PDMS) tubes, followed by analysis *via* thermal desorption (TD)-GC-MS (Kallenbach et al., 2014, 2015). One headspace VOC collection trial per week was performed from April to May 2020 on sunny days for 3 h between 11 am and 2 pm. Two to three plants of each chemotype with premature and mature (i.e., pollen presenting) flower heads were selected for each trial and placed 50 cm distant to one another in the greenhouse. The number of premature and mature flower heads (i.e., without and with pollen) was counted. Of each plant, 2–3 intact premature and 2–3 mature flower heads, of which no pollen had been harvested yet, were selected for VOC collection. The diameter of these flower heads was measured in each case to later normalize the measured compound abundances to the summarized flower head surface enclosed in VOC collection units (Supplementary Figure S1). The units were polypropylene cups of 50 mL volume (Premium Line, Tedeco-Gizeh, D), which were fixed with steel wire on wooden sticks at the exterior of the respective plant pots. The units were closed with respective lids containing holes of Ø 15 mm, through which the selected flower heads were gently threaded. The holes prevented heating and waterlogging. Two VOC collection units were fixed on each plant individual, one for premature and one for pollen-producing flower heads. In addition, two collection units per trial were fixed on plant pots containing humidified substrate and served as control to identify contaminants originating from the pots, substrate or the setup. Prior to usage, the absorbent PDMS tubes (length 5 mm, external diameter 1.8 mm, internal diameter 1 mm; Carl Roth, D) were cleaned in 4:1 (v:v) acetonitrile:methanol and then heated to 230°C for 20 min in a helium flow of 60 mL min^–1^ (Kallenbach et al., 2014, 2015). Two PDMS tubes were carefully added to each VOC collection unit and remained in the floral headspace for 3 h. Afterward, the PDMS tubes were gently removed from the units and stored in separate air-sealed glass vials (1.5 mL) with polytetrafluoroethylene (PTFE) – inlet screw caps and sealed with PTFE tape at −20°C for max. 2 weeks until GC-MS analyses (see section “Measurements by GC-MS”).

### Measurements by GC-MS

Samples were analyzed by GC-MS (GC 2010plus – MS QP2020, Shimadzu, JP) in electron impact ionization mode, using a VF-5 MS column (30 m × 0.2 mm ID, 10 m guard column, Varian, United States) and helium as carrier gas. The GC settings were adjusted to maximize the resolution for the three different sample types. For heptane extracts of leaf tissue for chemotype determination, the column flow was 1.5 mL min^–1^. The GC-injection port was kept at 240°C and operated in a 10:1 split mode. The GC oven program started at 50°C for 5 min and increased to 280°C with 5°C min^–1^, which was hold for 5 min (total run duration 25 min). For hexane extracts of pollen for determination of terpenoid composition, the column flow was set to 1.5 mL min^–1^. The GC temperature program started at 50°C for 5 min and increased to 250°C at a rate of 10°C min^–1^ and to 300°C at a rate of 30°C min^–1^ with a hold time of 10 min (total run duration 37 min). Relative concentrations of terpenoids in leaf extracts and pollen washes were calculated by converting the obtained peak areas to sample dry weights and by normalizing the sample to the internal standard peak area (1-bromodecane). Floral headspace VOC were analyzed via TD on the same instrument (TD 30 – GC 2010plus – MS QP2020, Shimadzu, JP). Trapped VOC were desorbed from PDMS tubes at 230°C under a flow of 60 mL min^–1^ and adsorbed on a Tenax^®^ cryo-trap with a temperature of –20°C for 8 min. From the cryo-trap, the compounds were re-desorbed at 250°C for 3 min, transferred to the GC at 250°C in a 1:1 split mode, and migrated with a column flow of 1.6 mL min^–1^. The GC temperature program started at 50°C for 5 min and increased to 250°C at a rate of 10°C min^–1^ and to 280°C at a rate of 30°C min^–1^ with a hold time of 3 min (total run duration 29 min). For all GC-MS samples line spectra (30 – 400 m/z) of separated compounds were acquired in quadrupole MS mode. An alkane standard mix (C8–C20, Sigma Aldrich, D) was analyzed with the same respective method like the samples in order to calculate Kovats retention indices (KI) for targeted compounds (Kováts, 1958). Compounds were identified by comparing the RI and mass spectra with those of synthetic reference compounds, where available, with library entries of the National Institute of Standards and Technology NIST 2014, Pherobase (El-Sayed, 2012), and mass spectra reported in Adams (2007). Compound quantification was based on the total ion chromatogram of peaks. For floral headspace samples, control samples (VOC collection units without flowers), and blanks (cleaned PDMS tubes) were used to identify and subtract contaminations.

### Florivorous Beetles and Four-Field Olfactometer Choice Assay

*Olibrus* spp. (Phalacridae) beetles occur very frequently in Northern Germany on yellow flowering Asteraceae (Müller-Schärer and Brown, 1995). For bioassays, *Olibrus* spp. were collected from *T. vulgare* and *Taraxacum officinale* flower heads in August and September 2019 and April to June 2020 from two sites. The first site was close to Gütersloh (Germany, 51°52′45.2′N, 8°17′04.6′E, elevation 68 m), the second close to Bielefeld University (Germany, 52°03′07.7′N, 8°28′43.0′E, elevation 105 m). The beetles were kept in boxes with mesh lids and fed with purchased pollen (Buxtrade, D). *Olibrus* spp. determination to species-level is difficult and beetles are thereby harmed. The species was hence determined on a subset of ≥*N* = 15 beetles from each site after finishing the bioassays following the protocol described in Freude et al. (1967) and individuals could be assigned to two species, *Olibrus affinis* (Sturm, 1807) (Gütersloh approximately 67%, Bielefeld approximately 47%) and *Olibrus millefolii* (Paykull, 1800) (approximately 33 and 53%, respectively). Four premature and four mature flower heads were cut from greenhouse-grown plants (see section “Plant Cultivation, Chemotype Determination and Selection”) for the florivore choice bioassays and placed upright on ice in Parafilm-sealed Petri dishes lined with moist filter paper. Four-field olfactometers with 20 cm diameter and 4 cm height were used (Supplementary Figure S2, Kühnle and Müller, 2009). In each trial, one single premature and one mature flower head were placed in opposing olfactometer fields, each in a 2 cm diameter PET ring, covered with moist filter paper to exclude visual cues. The remaining two fields received empty PET rings with moist filter paper and served as control. Four different beetles were tested for four different flower head combinations of the same plant, placed alternating in all four olfactometer fields to account for bias due to position preferences. In each trial, a single adult beetle was placed on a permeable mesh surface above the test fields, which was covered with a glass pane about 1 cm above the mesh. The beetle’s position (i.e., olfactometer field) was recorded every 10 s for 3 min. Non-responders, i.e., beetles that did not move at all within the 180 s of a trial, were excluded. Data for the four tested beetles per plant individual were averaged prior to statistical analyses and beetles were only used once per trial.

### Semi-Field Experiment: Florivore Abundance and Flower Head Miner Counts

Abundance of florivorous beetles on *T. vulgare* flower heads was observed in August 2012 and flower head miners were counted in November 2013 in the same semi-field experiment in Bielefeld Ummeln (see section “Plant Cultivation, Chemotype Determination and Selection”), which was planted in spring 2012. The field experiment site measured 6 × 55 m and was embedded within a meadow of extensively used agricultural grassland, surrounded by deciduous trees and shrubs. Spacing blocks of 6 × 11 m without intentionally planted *T. vulgare* divided the field into three blocks. Each block measured 6 × 11 m and contained four homogenous plots of each of four chemotypes and four heterogenous plots each containing all four chemotypes, i.e., eight plots of 1.5 × 1.5 m^2^ per block in total with randomly allocated individuals (see section “Measurements of Flower and Pollen Traits” for details). Heterogenous plots contained two plants of each of the four chemotypes. The chemotypes were β-thujone, *(E)-*carvyl acetate, (Z)-chrysanthenyl acetate and camphor (see section “Plant Cultivation, Chemotype Determination and Selection”). The eight plants in each plot had equal distance to each other and a minimum distance of 1 m to another plot or the site border. Sampling of adult florivores on flower heads was performed between 11 am and 2 pm on eleven days in August 2012. Each plant was observed for 2 min, avoiding casting a shadow on the observed plant. Example specimen were collected for determination to the species level (Chinery, 2004; Harde and Severa, 2006; Brohmer and Schäfer, 2010). Only a subset of *Olibrus* spp. individuals was sampled for determination and most specimens were scored in the field. Flower head infestation with miners was assessed in November 2013 on 10 flower heads of four plants per homogenous plot which were randomly chosen, resulting in a sample size of 120 flower heads for each of the four chemotypes. For this purpose, the seeds of the chosen flower heads were removed by hand and infested flower heads were counted.

### Statistical Analyses

The software ‘R’ version 4.0.3 (R Core Team, 2020) was used for statistical analyses. The number of produced flower heads per plant during the entire blooming period and the duration of the blooming period were analyzed with generalized linear mixed-effects models (GLMM) using a log-link Poisson distribution. To test for overdispersion, the dispersion of simulated residuals was compared to the observed residuals (function ‘simulateResiduals’ in package ‘DHARMa’ by Hartig, 2020). The amount of pollen per plant produced during the entire blooming period, the diameter of flower heads, pollen water content, and macronutrient contents (protein, carbohydrate, lipid, P:L-ratio) were analyzed with linear mixed-effects models (LMM) using an identity-link Gaussian distribution. Chemotype, the amount of pollen (if not used as response variable), and their interaction was included as fixed factors. The chemodiversity of compounds in the floral headspace and from pollen surface washes was assessed by both the compound richness (number) and calculating the Shannon index (Shannon and Weaver, 1949; function ‘diversity’ in package ‘vegan’ by Oksanen et al., 2015). The Shannon index is defined as *H* = -sum pi log(b) pi, where pi is in our case the proportional abundance of a compound i and b is the base of the logarithm. The Shannon-diversity was analyzed with an LMM using an identity-link Gaussian distribution and the compound richness (number) was analyzed using a GLMM with log-link Poisson distribution. All models included maternal genotype and the onset of blooming as random effects. We fitted the models with a maximum likelihood approach and applied step-wise backward model selection to obtain the minimal adequate model. Fixed effect terms with *P* < 0.05 were removed based on likelihood ratio tests (LRT). All (G)LMMs were performed using the (g)lmer function from the ‘lme4’ package (Bates et al., 2015).

Flower and pollen traits were correlated using Spearman’s rank correlation. Correlations between compound patterns of leaf extracts, pollen washes and floral volatile headspace samples were calculated by Mantel tests. For these analyses the dataset was reduced to those plant individuals of which all data were available (*N* = 28 plants) and the four datasets were transformed by Wisconsin double standardization, using the vegan package. Mantel tests were performed based on pairwise Kulczynski distances. Permutations were performed 10,000 times and Spearman rank correlations were used to compute Mantel’s *r* and *P*-values. Terpenoid profiles were compared between chemotypes within different types of samples (i.e., floral headspace, pollen, leaves) by using unsupervised Random Forest (RF) models (packages ‘randomForest’ and ‘party’ by Breiman, 2001). For each RF classification tree (the number of RF trees = iterations was set to 10,000), nine randomly selected variables were accepted as candidates at each split (mtry was set to 9 = approx. square root of the number of variables, i.e., the 82 compounds found across all samples). Multi-dimensional scaling of proximity matrix (MDS) plots were used to display RF model results. In addition, the compound abundances (normalized peak areas) detected in pollen washes were visualized as a heatmap (function ‘heatmap.2’ in package ‘gplots’ by Warnes et al., 2009).

The duration of stay above fields in the four-field-olfactometers (average percentage) of *Olibrus* spp. florivorous beetles were compared between flower types (=fixed factor) by LMM with identity-link Gaussian distribution within each of the chemotypes. More precisely, the proportional duration of stay above fields in the four-field-olfactometers to the headspace of premature flower heads (without pollen), mature flower heads (post anthesis, prior senescence) and control fields (without flower heads) was measured on four different beetles for each plant individual and the four observations per plant were averaged before LMM analyses. The plant individual was included as random factor in these models. Data were normally distributed, thus no data transformation was applied. Florivore abundance in semi-field experiments was compared between chemotypes (first fixed factor) in two plot types (second fixed factor: homogenous or heterogenous) and the interaction between these factors was included in the model. Floral head miner abundance in semi-field experiments was only counted in homogenous plots; thus, the chemotype was included as only fixed factor in these models. For both field-collected datasets GLMM with Poisson distribution were used and plot nested in block were included as random factors. Also for these models, backward model selection based on LRT was applied.

## Results

### Flower Traits, Bloom Period and Pollen Macronutrient Composition

**FIGURE 1:**
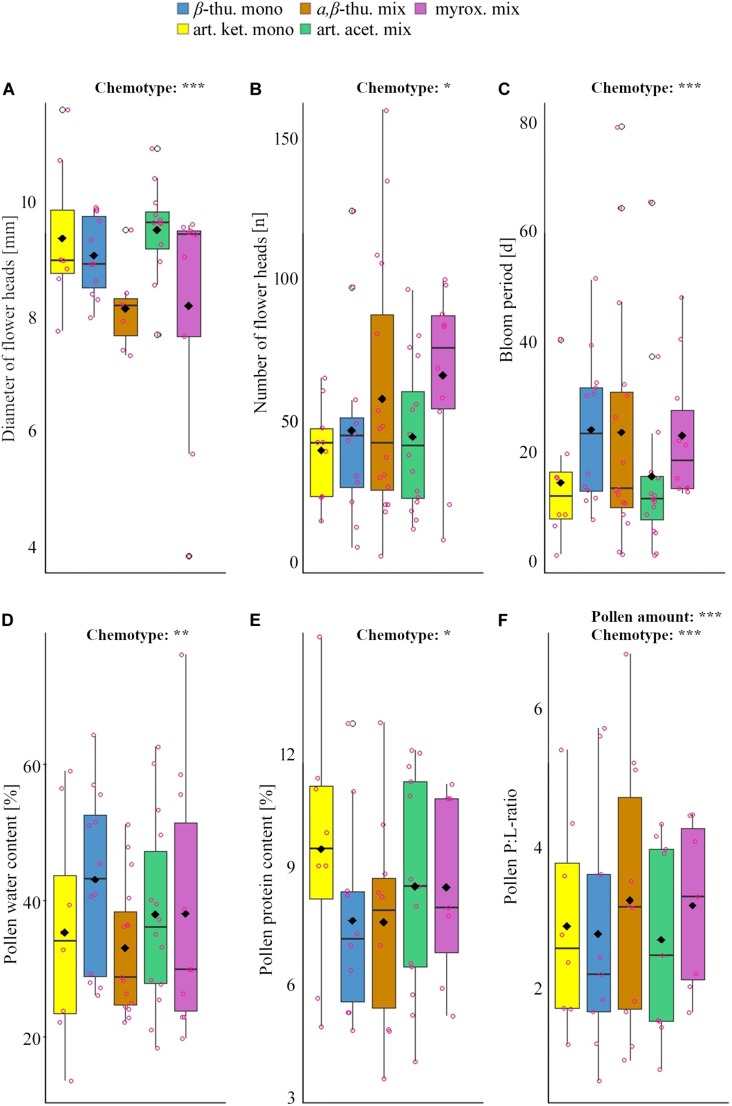
Significant differences in flower and bloom parameters **(A–C)** and pollen constituents **(D–F)** among different chemotypes of *Tanacetum vulgare*. Colors represent chemotypes (see legend). The boxplots show the median (horizontal line), mean (filled circle), first and third quartiles (hinges), and 1.5 of inter-quartile ranges (whiskers). Jitters (pink dots) display individual observations. Significance levels: ***P < 0.001; **P < 0.01; *P < 0.05. For detailed information on the statistical comparison of data, see Tables 1–3.

**FIGURE 2:**
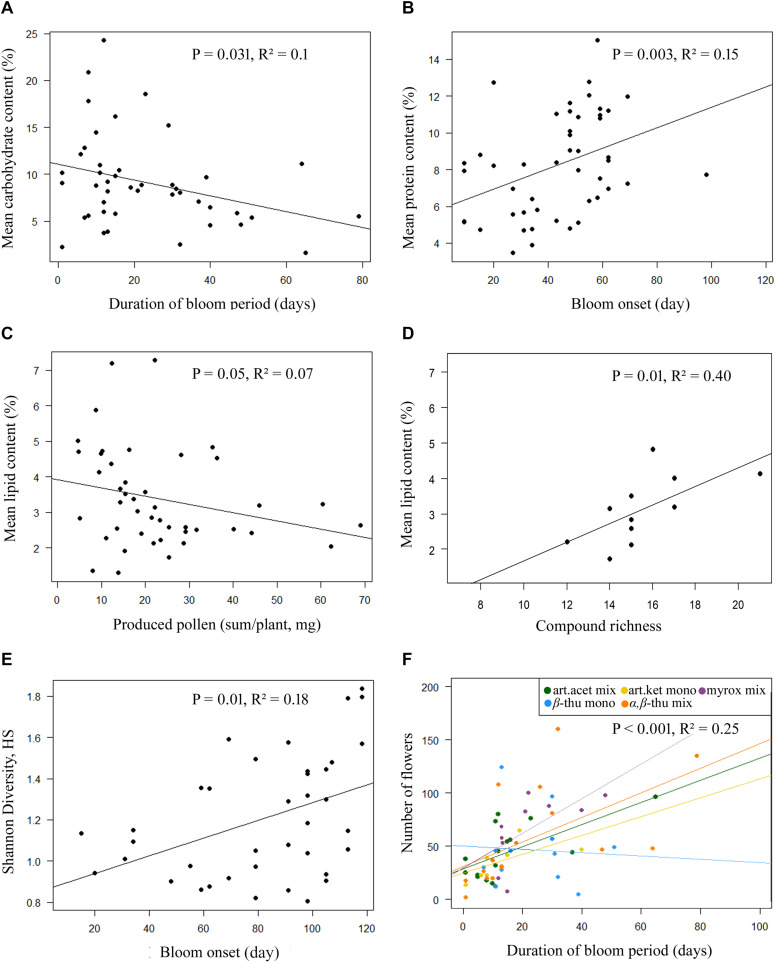
Linear regression plots for significant correlations of pollen macronutrient contents **(A–D)**, (Shannon) diversity or richness of detected compounds in pollen hexane surface washes **(D,E)**, and the number of produced flower heads per plant of *Tanacetum vulgare* **(F)** in relation to pollen and flower phenology parameters, namely the duration of the bloom period **(A,F)**, bloom onset **(B,E)**, the amount of produced pollen per plant **(C)**, and the number of detected compounds in pollen hexane surface washes **(D)**. In the plot **(F)**, the data for bloom period duration and number of flowers are displayed with different colors for chemotypes (see legend), as these data differed particularly strong between the chemotypes.

Greenhouse-grown plants of the five *T. vulgare* chemotypes differed significantly in the number of flower heads per individual over the entire blooming period, in the diameter of individual floral heads and in the duration of the blooming period (Tables 1, 2 and Figure 1). The myroxide mix-chemotype produced most flower heads but had smaller flowers compared to the other chemotypes. In contrast, the artemisia ketone mono-chemotype had fewer but larger flowers over a relatively short period. However, the number of flower heads was not correlated with floral diameter across all chemotypes (Spearman’s rank correlation, *P* = 0.53, parameter estimate = −0.12, *N* = 29). The bloom period was shortest in the artemisia acetate mix-chemotype and on average 40% longer for the α,β-thujone mix-chemotype. The amount of pollen per plant individual over the entire blooming period did not differ significantly between the five tested chemotypes. The macronutrient composition of pollen differed between chemotypes (Tables 1, 3 and Figure 1). There was a significant interaction of the factors chemotype and amount of pollen with the pollen protein content. On average, the pollen protein content was highest in the β-thujone mono-chemotype and lowest in the artemisia ketone mono-chemotype. Pollen of the chemotype α,β-thujone mix-chemotype had the highest protein-to-lipid (P:L) ratio, while pollen of the β-thujone mono-chemotype had the lowest P:L-ratio. Water, carbohydrate and lipid contents were not significantly different in the five tested chemotypes. The percentage of carbohydrates in pollen was negatively correlated with the duration of blooming period (Spearman’s rank correlation, *P* = 0.031, parameter estimate = −0.08, *N* = 44, Figure 2A). Pollen of later blooming flower heads contained higher proportions of protein (Spearman’s rank correlation, *P* = 0.003, parameter estimate = 0.06, *N* = 44, Figure 2B). Furthermore, the percentage of lipids in pollen was negatively correlated with the amount of pollen by each plant individual over the entire blooming period (*P* = 0.05, parameter estimate = −0.02, *N* = 44, Figure 2C). The number of detected compounds in pollen surface washes was not correlated with timing of flower onset (*P* = 0.77) but with pollen lipid content (*P* = 0.01, parameter estimate = 1.54, *N* = 11, Figure 2D). The Shannon diversity and compound richness in pollen washes did not differ significantly between chemotypes (Supplementary Table S2). However, the Shannon diversity increased as the blooming period progressed, i.e., later blooming flowers comprised a higher pollen Shannon diversity (*P* = 0.01, parameter estimate = 0.85, *N* = 37, Figure 2E). Individual plants produced flower heads for a period of up to 79 days and individuals with a longer bloom period had more flower heads (*P* < 0.001, parameter estimate = 0.24, *N* = 44, Figure 2F).

**TABLE 1:** Summary of (generalized) linear mixed-effects model analyses [(G)LMM] on traits of *Tanacetum vulgare* plants and behavioral responses of florivorous beetles toward *T. vulgare* flowers.

Response	Fixed effects	Random effects	Model	Model results	Model details
Flower heads (*n*)	CT	MG, BO	GLMM (Poisson, log link)	χ^2^ = 82.72, ***P* < 0.001**	Table 2
Ø flower heads (mm)	CT	MG, BO	LMM (Gaussian, identity link)	χ^2^ = 12.82, ***P* = 0.04**	Table 2
Bloom period (d)	CT	MG, BO	GLMM (Poisson, log link)	χ^2^ = 63.5, ***P* < 0.001**	Table 2
Pollen (mg)	CT	MG, BO	LMM (Gaussian, identity link)	χ^2^ = 3.92, *P* = 0.81	Table 2
Water (%)	CT × PA	MG, BO	LMM (Gaussian, identity link)	CT χ2 = 5.11, *P* = 0.32 PA χ2 = 7.98, ***P* = 0.007**	Table 3
Proteins (%)	CT × PA	MG, BO	LMM (Gaussian, identity link)	CTxPA χ^2^ = 13.97, ***P* = 0.02**	Table 3
Carbohydrates (%)	CT × PA	MG, BO	LMM (Gaussian, identity link)	CT χ^2^ = 4.09, *P* = 0.39 PA χ^2^ = 1.75, *P* = 0.19	Table 3
Lipids (%)	CT, PA	MG, BO	LMM (Gaussian, identity link)	CT χ^2^ = 5.46, *P* = 0.24 PA χ^2^ = 2.12, *P* = 0.15	Table 3
P:L-ratio	CT, PA	MG, BO	LMM (Gaussian, identity link)	**CT χ^2^ = 9604, *P* < 0.001 PA χ^2^ = 2765, *P* < 0.001**	Table 3
Pollen Shannon diversity (H′)	CT, PA	MG, BO	LMM (Gaussian, identity link)	CT χ^2^ = 2.17, *P* = 0.71 PA χ^2^ = 0.74, *P* = 0.39	Supplementary Table S2
Pollen compound richness (*n*)	CT, PA	MG, BO	GLMM (Poisson, log link)	CT χ^2^ = 1.17, *P* = 0.86 PA χ^2^ = 0.01, P = 0.7	Supplementary Table S2
Florivorous *Olibrus* spp. stay above olfactometer chamber (*n*)	FH	Plant individual	LMM (Gaussian, identity link), separate models for chemotypes	significant: β-thu. mono: χ2 = 389.4, ***P* < 0.001** art. acet. mix: χ2 = 219.8, ***P* = 0.002**	Supplementary Table S3
Florivorous *Olibrus* spp./plant (*n*)	CT × PT	plot:block	GLMM (Poisson, log link)	CT × PT χ^2^ = 13.1, ***P* = 0.002**	Supplementary Table S4
Flower mines/plant (*n*)	CT	plot:block	GLMM (Poisson, log link)	CT χ^2^ = 11.35, ***P* = 0.025**	Supplementary Table S4

*Significant *P*-values (*P* < 0.05) are highlighted in bold. If the interaction of fixed effects factors was not significant, the interaction was removed and the statistical test result is only shown for the single factors.*

*MG, maternal genotype; BO, bloom onset; CT, chemotype; PA, pollen amount; FH, flower head premature/mature/none; PT, plot type; H’, Shannon diversity index.*

**TABLE 2:** Differences in the number of flower heads, the diameter of mature flower heads, the duration of bloom period and the pollen production between plants of different *Tanacetum vulgare* chemotypes.

Response factor	Fixed effects	Parameter estimates ± SE for chemotypes					Variance estimates ± SE of random effects	Statistical test and result
		β-thu. mono	art.ket. mono	α, β-thu. mix	art. acet. mix	myrox. Mix		
Flower heads (n)	Chemotype	46.5 ± 9.4 *N* = 12	39.4 ± 10.9▼ *N* = 9	57.7 ± 8.1 *N* = 16	44.3 ± 8.1 *N* = 16	66 ± 10.3▲ *N* = 10	MG: 0.0 ± 3.4 (*g* = 12) BO: 0.0 ± 0.0 (*g* = 21)	GLMM (Poisson, log link) χ^2^ = 82.72, ***P* < 0.001**
Ø flower heads (mm)	Chemotype	9.2 ± 0.4 *N* = 11	9.4 ± 0.5▲ *N* = 7	8.2 ± 0.5 *N* = 7	9.4 ± 0.4▲ *N* = 12	8 ± 0.4▼ *N* = 9	MG: 0.6 ± 0.8 (*g* = 12) BO: 0.0 ± 0.1 (*g* = 18)	LMM (Gaussian, identity link) χ^2^ = 12.82, ***P* = 0.04**
Bloom period (d)	Chemotype	22.8 ± 4.7 *N* = 12	15.5 ± 5.8 *N* = 9	24.0 ± 4.2▼ *N* = 16	14.4 ± 4.2▼ *N* = 16	23.6 ± 5.1 *N* = 10	MG: 0.2 ± 0.5 (*g* = 12) BO: 0.7 ± 0.8 (*g* = 21)	GLMM (Poisson, log link) χ^2^ = 63.5, ***P* < 0.001**
Pollen (mg)	Chemotype	18.9 ± 4.0 *N* = 12	14.8 ± 4.6 *N* = 9	20.1 ± 3.5 *N* = 16	18.3 ± 3.5 *N* = 16	27.4 ± 4.3 *N* = 10	MG: 0.0 ± 0.6 (*g* = 12) BO: 0.0 ± 0.0 (*g* = 18)	LMM (Gaussian, identity link) χ^2^ = 3.92, *P* = 0.81

*Number of flower heads and pollen production are summed over the entire blooming period. Displayed are parameter estimates ± standard error values for the (generalized) linear mixed models [(G)LMM] and levels of significance for differences between chemotypes. Variance estimates are shown for the random effects maternal genotype (MG) and bloom onset (BO; i.e., the day on which the first mature flower was observed) and the number of groups of the respective random effect (g) is given in brackets. Significant values (*P* < 0.05) are highlighted in bold and pointing up ▲ or down ▼ triangles are added behind the highest and lowest parameter estimate, respectively.*

**TABLE 3:** Differences in pollen water and macronutrient contents between plants of different chemotypes of *Tanacetum vulgare*.

Response factor, pollen data	Fixed effects	Parameter estimates ± SE for chemotypes					Variance estimates ± SE of random effects	Statistical test and result
		β-thu. mono	art.ket mono	α, β-thu. mix	art. acet. mix	myrox. mix		
Water (%)	chemotype (CT), pollen amount (PA)	41.1 ± 4.5 *N* = 12	33.7 ± 5.4 *N* = 8	34.3 ± 4.1 *N* = 15	38.4 ± 4.28 *N* = 14	39.9 ± 4.7 *N* = 10	MG: 4.1 ± 6.4 (*g* = 12) BO: 0 ± 0 (*g* = 53)	LMM (Gaussian, ident link) CT × PA χ^2^ = 2.8, *P* = 0.69 CT χ^2^ = 5.11, *P* = 0.32 **PA χ^2^ = 7.98, *P* = 0.007**
Protein (%)	CT	9.1 ± 1.7▲	3.2 ± 2.5▼	5.6 ± 1.5	8.9 ± 1.3	6.3 ± 2.2	MG: 3.1 ± 5.6 (*g* = 11) BO: 4.6 ± 0.0 (*g* = 42)	LMM (Gaussian, ident link) **CT × PA χ^2^ = 13.97, *P* = 0.02**
	CT × PA	0.1 ± 0.1 *N* = 10	0.5 ± 0.1 *N* = 8	0.1 ± 0.1 *N* = 10	0.1 ± 0 *N* = 13	0.1 ± 0 *N* = 7		
Carbohydrates (%)	CT, PA	9 ± 1.8 *N* = 9	12.4 ± 1.8 *N* = 9	8.7 ± 1.8 *N* = 10	10.8 ± 1.9 *N* = 11	10.1 ± 2.4 *N* = 7	MG: 0 ± 0 (*g* = 11) BO: 1.3 ± 3.6 (*g* = 42)	LMM (Gaussian, ident link) CT × PA χ^2^ = 12.97, *P* = 0.09 CT χ^2^ = 4.09, *P* = 0.39 PA χ^2^ = 1.75, *P* = 0.19
Lipids (%)	CT, PA	4.1 ± 0.6	3.8 ± 0.5	3.3 ± 0.6	4.5 ± 0.6	3.5 ± 0.7	MG: 0.1 ± 0.3 (*g* = 11) BO: 0.2 ± 0.5 (*g* = 41)	LMM (Gaussian, ident link) CT × PA χ^2^ = 1.94, *P* = 0.77 CT χ^2^ = 5.46, *P* = 0.24 PA χ^2^ = 2.12, *P* = 0.15
		*N* = 9	*N* = 9	*N* = 10	*N* = 11	*N* = 7		
P:L-ratio	CT, PA	2.5 ± 0.4▼	3.1 ± 0.6	3.4 ± 0.5▲	2.8 ± 0.4	3.3 ± 0.6	MG: 0 ± 0 (*g* = 11) BO: 2.5 ± 1.6 (g = 40)	LMM (Gaussian, ident link) CT × PA χ^2^ = 5.07, *P* = 0.21 **CT χ^2^ = 9604, *P* < 0.001 PA χ^2^ = 2765, *P* < 0.001**
		*N* = 9	*N* = 8	*N* = 10	*N* = 9	*N* = 7		

*Displayed are parameter estimates and standard error values for the (generalized) linear mixed models [(G)LMM] and levels of significance for differences between chemotypes in pollen water, carbohydrate and lipid content (%) and the ratio of protein to lipid (P:L ratio). Random factors in all models were maternal genotype and bloom onset (i.e., the day on which the first mature flower was observed) and the number of groups for variance estimates is given in brackets. The total amount of produced pollen of each plant individual was included as fixed effect in models, to test factor interactions. The significance and parameter estimates for the factor pollen amount are shown in Table 2, thus not repeated here. Parameter estimates for the interaction of the fixed factors chemotype (CT) and pollen amount (PA) are only shown if significant. Variance estimates for the random effects maternal genotype (MG) and time of bloom onset (BO) are shown for the full model including the interaction, if the interaction was significant, otherwise the variance estimates refer to the minimized model, excluding the interaction. Significant values (*P* < 0.05) are highlighted in bold and pointing up ▲ or down ▼ triangles are added behind the highest and lowest parameter estimate, respectively.*

### Terpenoids and Other Organic Compounds in Floral Headspace and Pollen

Metabolic comparisons were conducted with greenhouse-grown *T. vulgare* plants of five different chemotypes. In total, surface washes of pollen contained 33 organic compounds (Supplementary Figure S3), compared to 63 compounds in leaf extracts, and 50 and 51 compounds detected in the headspace of premature and mature flower heads of the same *T. vulgare* plant individuals (Supplementary Table S1), respectively. In leaf extracts, the compounds with highest abundance were the compounds according to which the chemotypes were defined, i.e., particularly β*-*thujone in the β*-*thujone mono- and α, β-thujone mix-chemotypes and artemisia ketone in the artemisia ketone mono-chemotype and the artemisia ketone and artemisia acetate mix-chemotype, and the peak areas were highest for these compounds. Most abundant compounds in the headspace of mature, pollen-presenting flower heads were α*-*pinene and benzaldehyde, present in 94 and 91% of samples, respectively. In the headspace of premature flowers α*-*pinene and 3-hexen-1-ol-acetate occurred most frequently, in 97 and 87% of samples, respectively. Pollen washes contained in 87 and 89% of cases hexan-2-ol and *E*-3-hexenol, respectively (Supplementary Figure S3). Mantel tests revealed a correlation of the pollen compound pattern to that of the leaf extracts and the headspace of mature flower heads but not to the headspace compound pattern of premature flower heads (Table 4). Furthermore, the compound pattern of the leaf extracts correlated to the headspace of mature flower heads and headspace compound patterns for both ontogenetic stages of flowers also correlated. Random Forest comparisons of compound patterns did not reveal a clear separation for samples of the five chemotypes for floral headspace and pollen samples (Supplementary Figures S4A,B). Similarly, when comparing the compound abundances in a heat map (shown in Supplementary Figure S3 for pollen), no clear patterns for the different chemotypes were evident. In contrast, the compound patterns of leaf extracts clearly clustered (Supplementary Figure S4C).

**TABLE 4:** Mantel test comparisons within the same plant individuals of compounds detected in hexane pollen surface washes, leaf extracts and headspace samples for premature and mature flower heads.

	Pollen washes	Leaf extracts	HS premature flower
Leaf extract	***r* = 0.828 *P* < 0.001**		
Headspace (HS) premature flower	*r* = 0.1301 *P* = 0.152	*r* = 0.147 *P* = 0.107	
Headspace (HS) mature flower	***r* = 0.372 *P* < 0.001**	***r* = 0.373 *P* < 0.001**	***r* = 0.21 *P* = 0.032**

*N = 28 plants, merged dataset for the five chemotypes used in laboratory experiments. Significant values (*P* < 0.05) are highlighted in bold.*

### Florivore Preferences (Four-Field-Olfactometer Assay) and Abundance of Florivorous Beetles and Mines on Flower Heads (Field)

**FIGURE 3:**
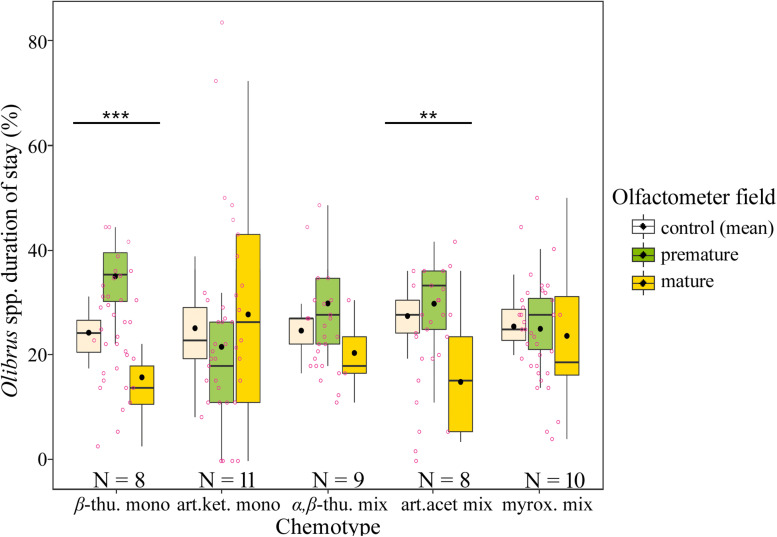
Duration of stay of florivorous beetles (*Olibrus* spp.) above olfactometer fields without flower head (control, mean values of both control fields), with premature flower head (prior anthesis), or with mature flower head (post anthesis) of five *Tanacetum vulgare* chemotypes. Four beetle trials were conducted for each plant individual and data were averaged to the plant individual level prior display and statistical comparison to avoid pseudoreplication. The boxplots show the median (horizontal line), mean (filled circle), first and third quartiles (hinges), and 1.5 of inter-quartile ranges (whiskers). Jitters (pink dots) display individual observations. Asterisks indicate significant differences between the three chambers (control, premature and mature flower heads). Significance levels of LMM: ***P < 0.001; **P < 0.01. For detailed statistical comparison of data, see Supplementary Table S3.

**FIGURE 4:**
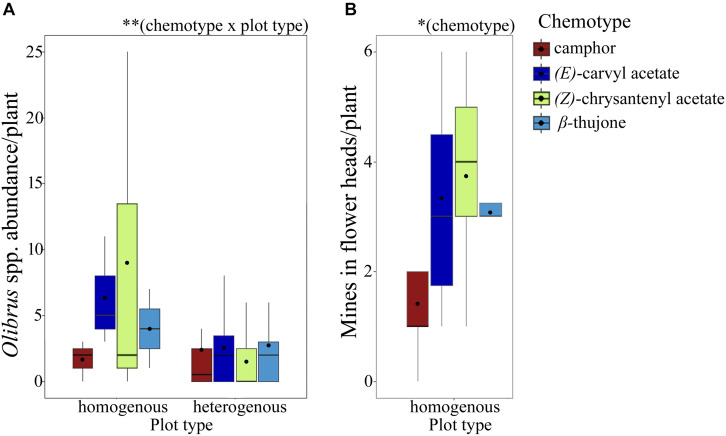
Abundance of florivorous *Olibrus* spp. beetles on flower heads (**A**, *N* = 18 plants per chemotype) and of mining insects in flower heads (**B**, *N* = 24 plants per chemotype) of four *Tanacetum vulgare* chemotypes in the field. Florivorous beetles were counted in plots containing only individuals of the same chemotype (homogenous plots) and in plots containing all four chemotypes (heterogenous plots). The boxplots show the median (horizontal line), mean (filled circle), first and third quartiles (hinges), and 1.5 of inter-quartile ranges (whiskers). Significance levels: ^∗^*P* < 0.05; ^∗∗^*P* < 0.01. For detailed statistical comparison of data, see Supplementary Table S4.

Florivorous beetles (*Olibrus* spp.) were tested for their response to headspace volatiles of flower heads from greenhouse-grown plants. For two of the five tested chemotypes, namely β-thujone mono- and artemisia acetate mix-, the beetles spend a significantly longer time above fields containing premature flower heads compared to fields containing mature flower heads (Figure 3, Table 1, and Supplementary Table S3). The same trend was observed for the α,β-thujone mix-chemotype (*P* = 0.065). For the behavioral response toward flower head scent of the artemisia ketone mono-chemotype, the highest variances (standard error values) were recorded (Supplementary Table S3). Abundance of florivorous *Olibrus* spp. beetles on flower heads in the semi-field experiment depended significantly on the interaction of the plant chemotype with the plot type (Figure 4 and Supplementary Table S4). In homogenous field plots, the abundance of beetles was higher compared to the abundance on the same chemotypes in heterogenous plots. The abundance of mines (only observed in homogenous plots) depended significantly on the chemotype. The mean abundance of both adult beetles and floral head miners in homogenous plots was highest in the *(E)-*carvyl acetate and (Z)-chrysanthenyl acetate mono-chemotypes, while plants of the camphor mono-chemotype were least frequently visited.

## Discussion

### Patterns and Diversity in Flower and Pollen Scent Compounds

We had hypothesized that *T. vulgare* plants express chemotype-specific patterns of VOC in the floral headspace and pollen, but this hypothesis could only partially be supported by our data. Random Forest analyses revealed no clustering into chemotype-specific groups for floral headspace or pollen washes (Supplementary Figures S4A,B), in contrast to the clear clustering found for leaf extract compounds (Supplementary Figure S4C). Thus, the overlap of scent compounds in the floral headspace and pollen among the different chemotypes may distract pollinators and ensure that they visit all chemotypes and transfer pollen from one chemotype to another. In line with this hypothesis, a recent study revealed that honeybees did not discriminate between pollen scents if the pollen had a different nutritional value and/or taxonomic origin, as long as the respective plants had an overlapping bloom period (Pietrantuono et al., 2019). In *T. vulgare*, we found that surface washes of pollen contained only approximately half of the number of organic compounds detected in leaf extracts, but we cannot exclude that this difference is at least in part due to the different analytical methods for both sample types. Within flowers, a tissue-specific emission of volatiles is a characteristic feature of many species. In general, petals are the primary source of floral volatiles, although other tissues (stamens, pistils, sepals, and nectaries) also contribute to the floral bouquet in certain plant species (Dobson et al., 1997; Dötterl and Jürgens, 2005; Flamini et al., 2007). Moreover, pollen are often characterized by distinct scent emission profiles, as for instance found in *Citrus limon* (Rutaceae) (Flamini et al., 2007) and *Ranunculus acris* (Ranunculaceae) (Bergström et al., 1995). While the compound patterns of pollen and pollen-producing flowers showed similarities to those of leaves in our investigation of *T. vulgare*, premature flowers differed in this regard. The opposite pattern regarding floral ontogeny and scent has been described for the shrub *Asimina triloba* (Annonaceae). Premature flowers of this species produce the same sesquiterpenoids as the leaves, but mature flowers emit a specific scent of fermentation volatiles (Goodrich et al., 2006). However, literature on changes in flower scent emission in the course of floral ontogeny is scarce and the example of *A. triloba* may not be representative for various other plant species.

Interestingly, terpenoid patterns also vary between above- and belowground tissues in our study plant *T. vulgare*. In the roots sesquiterpenoids dominate, whereas in the shoots mainly monoterpenoids are expressed (Kleine and Müller, 2014), indicating independent terpenoid biosynthesis in different tissues within plants of this species. The composition of the individual compounds is partly different between the tissues, and we addressed the question, whether the number or diversity of the individual compounds between the tissues in the flowers is related. In this context, for deterrent and repellent compounds in floral headspace volatiles (Raguso, 2009) and for toxins in reward compounds (Rivest and Forrest, 2020), the pleiotropy hypothesis has been proposed and controversially discussed. The hypothesis explains the evolution of the presence of floral defensive compounds by a random physiological spill-over effect of these compounds from other plant tissues in which they originally evolved as protection agents. We found no correlation between compound patterns in leaves and premature flowers but between leaves and mature flowers. However, our results support the pleiotropy hypothesis for the presence of compounds in *T. vulgare* leaves and floral tissues to some degree: Of the detected compounds approx. one third were present in all four types of samples, i.e., headspace samples of premature and mature flower heads, pollen washes and leaf extracts, indicating an overlap and potential spill-over in the metabolic composition (Supplementary Table S1).

Furthermore, we addressed the question, if a higher compound diversity trades off with the resource-demanding production of pollen. We found a higher Shannon diversity of pollen surface compounds and a higher pollen protein content in later blooming flowers, which contradicts our trade-off hypothesis. Thus, we propose that the reduction of pollinators at the end of the flowering season of *T. vulgare* (Dupont et al., 2018) leads to a greater need to attract the still available pollinators for late-blooming flowers, and thus pollinators are lured with higher protein levels in pollen. The higher pollen compound diversity later in the blooming season may provide a better defense of the pollen against robbery (Celedon and Bohlmann, 2019). Under natural conditions, in addition also changes in temperatures and in the occurrence of drought events later in the season may affect the composition and chemodiversity of flower volatiles (Farré-Armengol et al., 2020). We expected plant individuals with a high floral headspace chemodiversity to produce less pollen, potentially due to higher enzymatic costs. The compound diversity and pollen amount were, however, not directly linked. Estimations of costs for the production of metabolites are difficult, as most of the detected compounds in the floral headspace and in pollen washes were terpenoids, of which one single enzyme can produce multiple different ones but for some terpenoids also multiple enzymatic steps are needed (Gershenzon, 1994). Thus, a higher compound diversity is not necessarily linked to higher costs.

### Flower Production and Pollen Macronutrients

Significant differences were found in the number and diameter of flower heads and the duration of blooming period between the five *T. vulgare* chemotypes investigated in laboratory and greenhouse experiments. The lowest number of flower heads with the largest diameter and a relatively short blooming period was observed for the artemisia ketone mono-chemotype (Table 2) and this chemotype produced only few pollen with the lowest water and protein content. Former studies showed that the number of flower heads is positively correlated with attractiveness for pollinators in the Asteraceae *Jacobaea vulgaris* (formerly: *Senecio jacobaea*) (Andersson, 1996), *Achillea ptarmica* (Andersson, 1991), and *Pertya triloba* (Kawarasaki and Hori, 1999). In a field collection of 140 plants at the same site where *T. vulgare* seeds for the present study were collected, the artemisia ketone mono-chemotype was the second most frequently represented, thus seemingly a very successful and highly competitive chemotype (Kleine and Müller, 2011). However, it remains to be elucidated if the larger flower size of the artemisia ketone mono-chemotype found in the present study attracts more pollinators or even only certain pollinator types under field conditions and can thus compensate for the low amount of flowers, pollen and pollen protein in this chemotype. Instead, this chemotype may have multiple blooming periods or invest rather in vegetative reproduction, which may explain its success in the field. But since there are hardly any extensive field studies on trophic interactions with chemotypes, this remains to be a topic for future analyses. In fact, vegetative reproduction by ramets is an important growth strategy of this perennial species (Kleine et al., 2017). The shortest bloom periods were observed for the artemisia ketone and artemisia acetate mix-chemotypes, but within this short period, plants of these chemotypes produced the highest numbers of flower heads (Figure 2F). These examples highlight intraspecific differences in the strategies for pollinator attraction and reproduction among the different chemotypes, which is a novel and particularly interesting finding.

We expected the proportions of pollen macronutrients to differ between the chemotypes and trade-off with the blooming duration (i.e., fewer nutrients, longer bloom period) and the amount of produced pollen per plant individual (i.e., more nutrients, lower amount of produced pollen). We did indeed find chemotype-related differences in the proportions of pollen macronutrients, i.e., water, protein and P:L-ratio. Moreover, trade-offs between pollen macronutrients and blooming parameters were detected, which suggest resource limitations in the production of pollen. In particular, plants with a shorter blooming period showed higher carbohydrate contents than plants with a longer blooming period. Pacini (1996) found differences in the pollen carbohydrate composition between dehydrated and non-dehydrated pollen and postulated that cytoplasmic carbohydrates and sucrose are protection-agents of pollen and maintain viability during exposure and dispersal. Pollen contains various sources of carbohydrates, such as starch and mono- and polysaccharides such as callose, with different function (Pacini, 1996), and further studies are required to elucidate, if only the carbohydrate contents, or also the composition differs and how differences in carbohydrate content and composition affect pollen viability and flower visitation at different time points within the bloom period. Regarding protein, previous studies of pollen macronutrient contents showed that pollen ranges from 1.5 to 61% protein by dry mass across different plant species (Roulston et al., 2000; Vaudo et al., 2020). In our intraspecific comparison, protein contents in pollen ranged from 3 to 15% between different chemotypes, with 8% on average, and similar protein contents were reported from pollen of other Asteraceae plants (Nicolson and Human, 2013; Vaudo et al., 2020). Pollen protein is supposed to be the most important macronutrient for bees; across 68 bee and six plant species, significant correlations between pollen protein content, bee abundance and visitation rate were found (Russo et al., 2019). Similarly, the P:L-ratio could be directly related to floral attractiveness for bumble bee workers, with P:L-ratios of 5:1 and 10:1 being most attractive (Vaudo et al., 2016). Thus, according to the protein content and P:L-ratios, *T. vulgare* flowers should be poorly rewarding and not very attractive to bees and other pollinators. However, *T. vulgare* flowers are visited by various generalist and specialized pollinating insects of different species and families, such as dipterans (Peach and Gries, 2016), hymenopterans (Müller and Kuhlmann, 2008; Praz et al., 2008a), and lepidopterans (Bąkowski and Boroń, 2005).

In addition to macronutrients, other constituents of reward traits may be of value for flower visitors. For instance, pollen of Asteraceae can protect bees from brood parasitism (Spear et al., 2016) and terpene-rich pollen of Lamiaceae helps bees to fight off pathogens (Wiese et al., 2018). Moreover, we found very high amounts of pollen of up to 70 mg fresh weight per individual in *T. vulgare* and individual plants flowered up to 80 days, which may compensate for the non-optimal nutrient composition. Pollen constituents reward and attract pollinators, but specialized metabolites in rewards also mediate interactions with microbes (Huang et al., 2012; McArt et al., 2014) and pollen proteins and lipids are essential for pollen tube growth (Wolters-Arts et al., 1998; Ruedenauer et al., 2019). Thus, future research is required to clarify whether the differences in pollen macronutrient contents of *T. vulgare* chemotypes affect interactions with pollinating insects and microbes and whether the pollen nutrient content trades-off with the ability of the pollen to germinate and penetrate the stigma.

### Florivore Attraction and Abundance

We hypothesized that florivorous beetles prefer volatile cues of mature flower heads to those of unripe flower heads, but we found the opposite effect in olfactometer assays, in which visual cues were hidden. This result indicates that florivorous beetles discriminate between the odors of flowers of different ontogenetic stages. While collecting the beetles in the field, we found them on mature and premature flowers. However, so far there are no available field studies describing at which maturation stage of the flowers the beetles settle at the beginning of the season and whether beetle preferences change over the season. Mature flowers should be particularly attractive to the beetles after birth or hibernation, because they provide pollen as food source. Premature flowers may be particularly attractive for beetles with reproductive pressure, as the females lay the eggs near immature seeds. Our results suggests that pollen is emitting repellent compounds to the beetles. However, the mature flower heads were tested in our experiments shortly after anthesis. Whether the floral scent of mature *T. vulgare* flowers becomes attractive again to the beetles with continuing maturity remains to be tested. Moreover, our field abundance data of florivorous beetles revealed preferences for certain chemotypes. These results support our hypothesis and are in line with previous studies showing similar distinct chemotype-specific preferences for foliar herbivores, such as beetles (Wolf et al., 2012b), or aphids (Völkl et al., 1999; Kleine and Müller, 2011; Benedek et al., 2015). However, we exclusively tested mono-chemotype plants. It will be interesting to compare the attractiveness of mono- and mix-chemotype plants toward flower visitors and we expect preferences for flower heads of mix-chemotype plants due to lower concentrations of individual compounds in headspace volatiles (Müller et al., 2020).

In our semi-field experiment we expected chemotype-specific differences in florivore abundance to diminish in heterogenous stands with different chemotype neighbors due to overlapping odor plumes (Riffell et al., 2014; Cai et al., 2017), increased chemical diversity of the scent bouquet per plot and a dilution of the headspace concentration and/or masking of single repellent or deterrent compounds. Our field data support this hypothesis and indicate that heterogenous plots are on average less infested by florivores. However, an additional reason besides altered odor plumes may explain the differences in beetle abundance: In our semi-field experiment, we observed differences in the flowering time of individual chemotypes, similarly to the differences that we found for our greenhouse-grown plants. Thus, in some of the homogeneous plots more flowers may have been present simultaneously, while in none of the heterogeneous plots all plants were in full bloom at the same time.

## Conclusion

In conclusion, we showed that *T. vulgare* chemotypes differ remarkably in flower traits such as flower head diameter, bloom period and pollen P:L-ratio. Florivorous beetles were able to discriminate between flower heads in different ontogenetic stages by scent. In a semi-field experiment, the beetles showed clear chemotype-specific preferences but their choice was distracted by the presence of different chemotype neighbors. These results provide insight to the chemical ecology of a plant species with fascinating intraspecific chemodiversity and indicate that metabolic and ecologic traits are highly linked.

## Data Availability Statement

The data were now uploaded to the Knowledge Network for Biocomplexity (KNB): https://knb.ecoinformatics.org/view/urn%3Auuid%3A1cb33aae-b19e-4b48-8fae-96f00c2e354a.

## Author Contributions

CM, EE, and SK conceived and designed the experiments. CM and EE acquired the funding. SW, SE, and SK performed the semi-field experiments and analyzed the respective data with the help of CM. EE performed the experiments with greenhouse-grown plants, i.e., chemical analyses and olfactometer assays, analyzed respective data, conducted all statistical analyses, prepared all figures and tables, and wrote the first version of the manuscript. All the authors revised and commented on the manuscript.

## Conflict of Interest

The authors declare that the research was conducted in the absence of any commercial or financial relationships that could be construed as a potential conflict of interest.
