# Supplementary material for: Flower Production, Headspace Volatiles, Pollen Nutrients, and Florivory in Tanacetum vulgare Chemotypes
Source: Front Plant Sci. 2021 Jan 20;11:611877. doi: 10.3389/fpls.2020.611877 (PMC7855176; doi:10.3389/fpls.2020.611877)
Supplement: Supplementary file 1 [file Data_Sheet_1.docx]

**Supplementary data**

**Flower production, headspace volatiles, pollen nutrients and florivory in *Tanacetum vulgare* chemotypes**

Elisabeth J. Eilers^1*^, Sandra Kleine^1^, Silvia Eckert^1,2^, Simon Waldherr^1^, Caroline Müller^1^

^1^Chemical Ecology, Bielefeld University, Bielefeld, Germany

^2^Biodiversity Research/Systematic Botany, University of Potsdam, Potsdam, Germany

*** Correspondence:**Elisabeth J. Eilers
elisabeth.eilers@uni-bielefeld.de


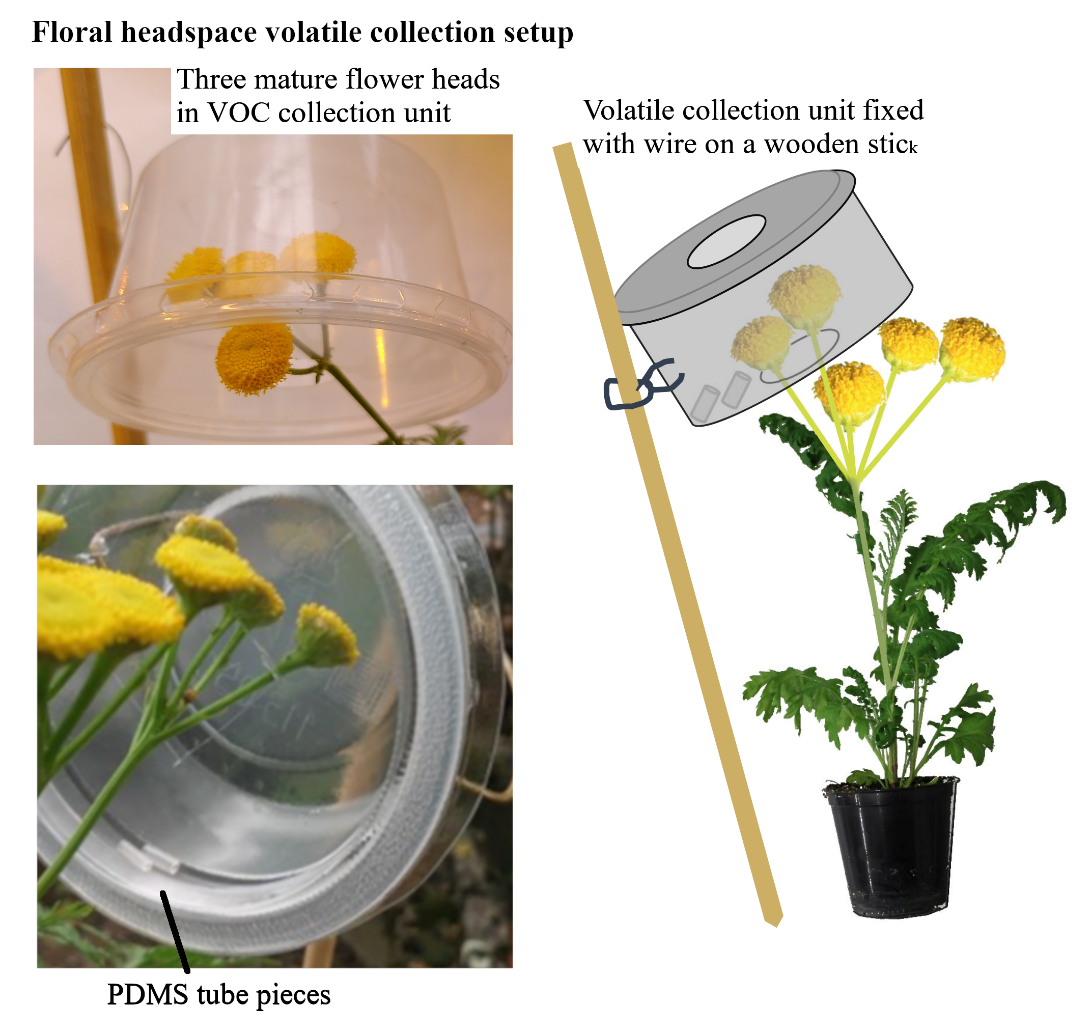


**Fig. S1. Photographs and schematic drawing of the floral headspace volatile collection setup.** Two to three intact flower heads were gently threaded through a Ø 15 mm circular opening in a VOC collection unit, i. e. a 50 mL polypropylene cup with lid. An additional hole in the lid of the same diameter prevented heating and waterlogging. Two VOC collection units were fixed on the same plant individual, one for premature and one for pollen-producing flower heads. Headspace volatiles were trapped on PDMS (i.e. silicone) absorbent tubes and analysed by TD-GC-MS.


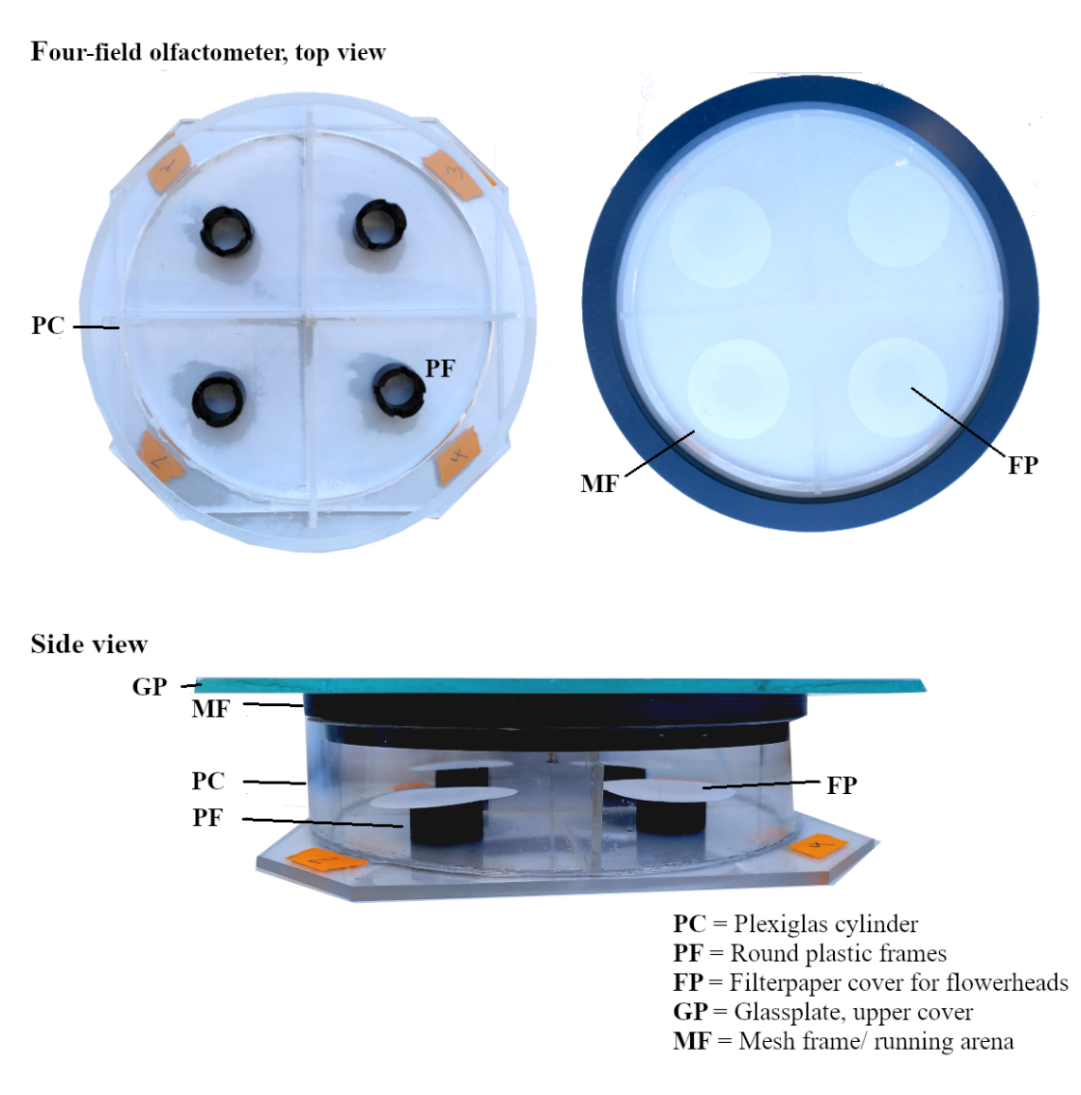


**Fig. S2. Photographs of the used four-field olfactometer for choice assays with *Olibrus* spp. and *T. vulgare* flower heads.** The plexiglass cylinder was 20 cm in diameter and 4 cm high. In order to avoid visual cues, the flower heads were placed in round plastic frames (PF) and covered with filter paper (FP). The beetles walked on a mesh frame (MF) on top of the two test and two control fields, which served as running arena and was covered with a glass plate (GP).

**
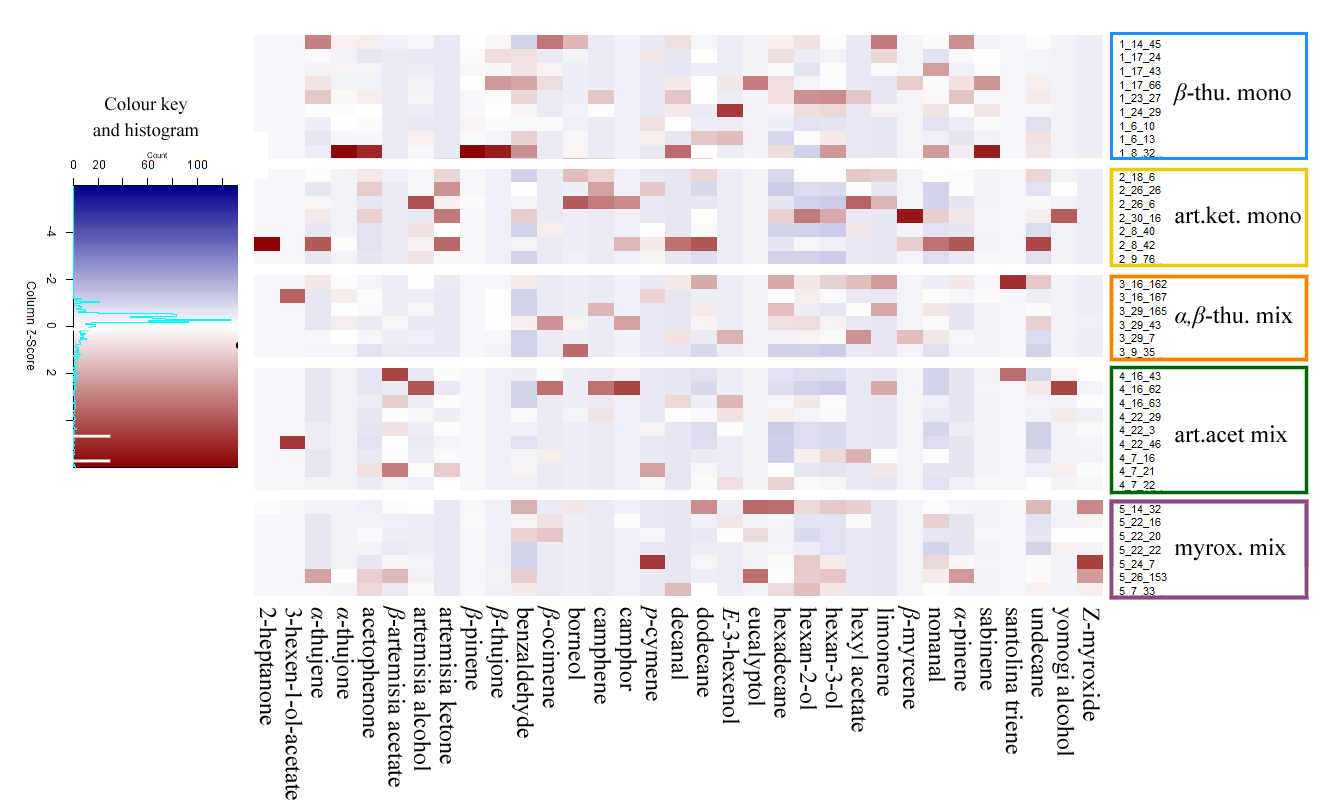
**

**Fig. S3. Heat map of organic compounds detected in surface washes of pollen of five chemotypes of *T. vulgare*.** Each row represents one plant individual, ordered by chemotypes, each column comprises one compound in alphabetical order. The colours from blue to red represent the Z-score (see legend), which is a measure of distance, in standard deviations, from the mean of samples.


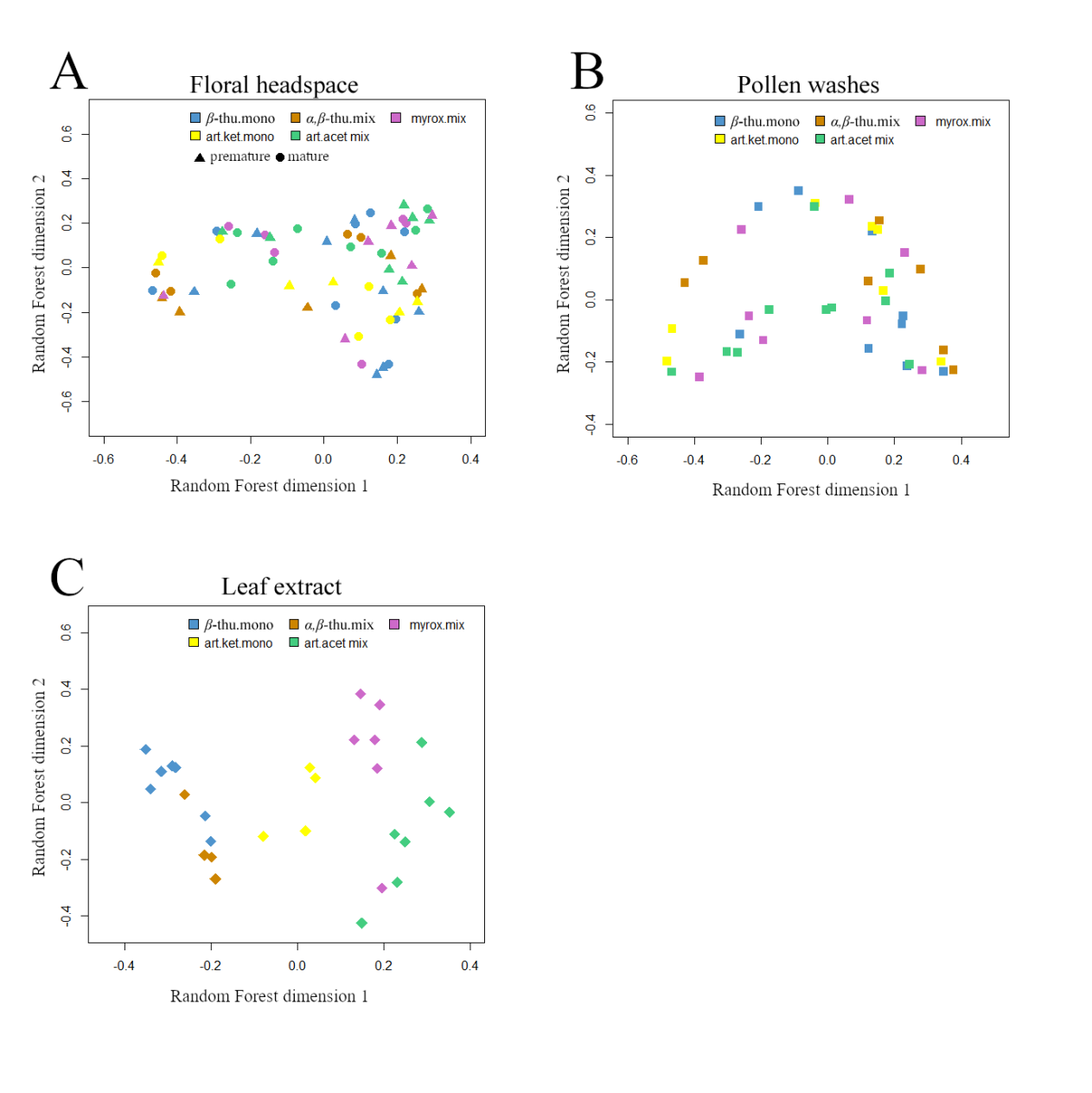


**Fig. S4. Unsupervised Random Forest comparisons of organic compounds detected in headspace collections from premature and mature flower heads (A), in surface washes of pollen (B) and in leaf extracts (C) of five chemotypes of *T. vulgare*.** Colours represent chemotypes and symbols represent sample type (see legend).

Table S1. Identification of volatile organic compounds detected in the floral headspace of premature and mature *T. vulgare* flower heads, and in leaf and pollen heptane extracts of the same plants. The value of the highest molecular ion peak (m/z) for each detected compound (=base peak) is listed, together with Kovats retention indices (KI) and the percentage of the detected peak area, averaged among samples and within the sample type.

|  |  |  |  |  | Percentage of peak area within sample type | | | |
| --- | --- | --- | --- | --- | --- | --- | --- | --- |
| Compound no. | Compound | m/z | KI | mature flower head | premature flower head | leaf extract | pollen extract | present in all four sample types |
| 1 | hexan-3-ol | 59 | 797 | <0.01 | <0.01 | - | <0.01 |  |
| 2 | hexanal | 44 | 799 | 0.06 | 0.16 | - | - |  |
| 3 | hexan-2-ol | 45 | 808 | - | - | 47.10 | 55.24 |  |
| 4 | *E*-3-hexenol | 41 | 858 | 0.85 | 1.00 | 23.58 | 26.97 | x |
| 5 | heptanal | 44 | 899 | 0.21 | 0.11 | - | - |  |
| 6 | santolina triene | 93 | 902 | 0.13 | 0.06 | 0.54 | <0.01 | x |
| 7 | *α*-thujene | 93 | 930 | 2.14 | 2.60 | 0.04 | 0.05 | x |
| 8 | *α*-pinene | 93 | 937 | 24.63 | 38.38 | 0.07 | 0.05 | x |
| 9 | 2-heptanone | 43 | 944 | 8.09 | 4.81 | 2.13 | 2.50 | x |
| 10 | camphene | 93 | 954 | 2.17 | 2.95 | 0.03 | <0.01 | x |
| 11 | benzaldehyde | 106 | 969 | 14.86 | 2.79 | 0.18 | 0.20 | x |
| 12 | sabinene | 93 | 977 | 0.48 | 0.18 | 0.53 | 0.24 | x |
| 13 | *β*-pinene | 93 | 982 | 0.41 | 0.19 | 0.19 | 0.19 | x |
| 14 | *β*-myrcene | 93 | 991 | 0.21 | 0.24 | <0.01 | <0.01 | x |
| 15 | *Z*-myroxide | 79 | 994 | 3.97 | 3.32 | 2.13 | 0.67 | x |
| 16 | yomogi alcohol | 43 | 996 | 0.01 | 0.09 | 0.16 | 0.05 | x |
| 17 | decane | 43 | 1000 | 0.46 | 0.88 | <0.01 | - |  |
| 18 | *Z*-3-hexenyl acetate | 43 | 1005 | 6.27 | 6.04 | 0.06 | 0.07 | x |
| 19 | hexyl acetate | 43 | 1012 | 0.22 | 0.19 | - | <0.01 |  |
| 20 | *o*-cymene | 119 | 1018 | - | - | 0.21 | - |  |
| 21 | *α*-terpinene | 93 | 1021 | 0.10 | 0.04 | - | - |  |
| 22 | *p*-cymene | 119 | 1030 | 1.11 | 1.54 | 0.01 | 0.02 | x |
| 23 | limonene | 68 | 1034 | 1.46 | 2.07 | 0.01 | 0.01 | x |
| 24 | 1,8-cineole | 43 | 1040 | 1.38 | 1.16 | 0.24 | 0.03 | x |
| 25 | benzyl alcohol | 79 | 1041 | 1.24 | 0.76 | - | - |  |
| 26 | *β*-ocimene | 93 | 1050 | 0.04 | 0.31 | 0.01 | 0.01 | x |
| 27 | artemisia ketone | 83 | 1061 | 2.25 | 4.52 | 3.91 | 0.99 | x |
| 28 | 2-methyldecane | 43 | 1063 | - | - | 1.80 | 2.11 |  |
| 29 | *γ*-terpinene | 93 | 1064 | 0.91 | 1.24 | 0.08 | - |  |
| 30 | acetophenone | 105 | 1073 | 5.13 | 3.03 | 0.53 | 0.61 | x |
| 31 | *E*-sabinenhydrate | 43 | 1077 | 0.13 | 0.05 | 0.41 | - |  |
| 32 | artemisia alcohol | 85 | 1083 | 0.72 | 0.90 | 0.90 | 0.25 | x |
| 33 | hexanoic acid | 60 | 1085 | 1.75 | 1.83 | - | - |  |
| 34 | undecane | 57 | 1097 | 0.93 | 1.46 | 2.03 | 2.30 | x |
| 35 | nonanal | 57 | 1108 | 3.71 | 3.53 | 0.81 | 0.92 | x |
| 36 | *Z*-sabinene hydrate | 43 | 1110 | - | - | 0.16 | - |  |
| 37 | *α*-thujone | 110 | 1113 | 4.77 | 4.26 | 1.71 | 1.98 | x |
| 38 | *β*-thujone | 110 | 1125 | 3.37 | 3.22 | 3.20 | 2.35 | x |
| 39 | chrysanthenon | 81 | 1129 | - | - | 0.06 | - |  |
| 40 | isopinocarveol | 41 | 1132 | 0.01 | - | - | - |  |
| 41 | unknown 1 | 107 | 1133 | - | - | 0.02 | - |  |
| 42 | *Z*-sabinol | 92 | 1149 | 0.08 | 0.03 | 0.08 | - |  |
| 43 | camphor | 95 | 1158 | 0.13 | 0.18 | 0.08 | - |  |
| 44 | isothujol | 43 | 1159 | 0.20 | 0.06 | - | - |  |
| 45 | *β*-artemisia acetate | 85 | 1165 | 1.20 | 2.37 | 2.09 | 0.71 | x |
| 46 | pinocarvone | 108 | 1166 | 0.23 | - | 0.10 | - |  |
| 47 | *E*-2-nonenal | 57 | 1168 | - | - | - | - |  |
| 48 | umbellulone | 108 | 1170 | 0.33 | 0.14 | 0.05 | - |  |
| 49 | verbenol | 81 | 1171 | - | - | - | - |  |
| 50 | *E*-chrysanthenol | 32 | 1175 | - | - | <0.01 | - |  |
| 51 | borneol | 95 | 1185 | 0.11 | 0.09 | 0.10 | 0.03 | x |
| 52 | dodecane | 57 | 1199 | - | - | 0.58 | 0.66 |  |
| 53 | decanal | 57 | 1209 | 0.64 | 1.13 | 0.06 | 0.07 | x |
| 54 | terpin-1-en-4-yl-acetate | 93 | 1221 | - | - | <0.01 | - |  |
| 55 | *Z*-*β*-chrysanthenyl acetate | 119 | 1238 | 0.28 | 0.01 | - | - |  |
| 56 | cumin aldehyde | 133 | 1240 | 0.15 | <0.01 | - | - |  |
| 57 | unknown 2 | 119 | 1248 | - | - | 0.05 | - |  |
| 58 | *E*-verbanol acetate | 93 | 1257 | - | - | <0.01 | - |  |
| 59 | α-chrysanthenyl acetate | 43 | 1265 | 0.38 | 0.03 | 0.09 | - |  |
| 60 | hexadecane | 57 | 1270 | - | - | <0.01 | 0.68 |  |
| 61 | nonanoic acid | 60 | 1280 | 1.72 | 1.25 | - | - |  |
| 62 | *Z*-sabinyl acetate | 91 | 1296 | - | - | 0.14 | - |  |
| 63 | undecanal | 43 | 1312 | 0.07 | 0.19 | - | - |  |
| 64 | *β*-carvylacetat | 84 | 1331 | - | - | - | - |  |
| 65 | eugenol | 164 | 1367 | - | - | 0.10 | - |  |
| 66 | dodecanal | 41 | 1410 | 0.33 | 0.51 | - | - |  |
| 67 | *β*-caryophyllene | 93 | 1443 | 0.04 | 0.01 | 0.04 | - |  |
| 68 | unknown 3 (sesqui) | 43 | 1454 | - | - | 0.15 | - |  |
| 69 | unknown 4 (sesqui) | 57 | 1483 | - | - | 0.01 | - |  |
| 70 | unknown 5 (sesqui) | 43 | 1494 | - | - | 1.59 | - |  |
| 71 | unknown 6 (sesqui) | 161 | 1504 | - | - | 0.07 | - |  |
| 72 | unknown 7 (sesqui) | 91 | 1508 | - | - | 0.02 | - |  |
| 73 | *α*-farnesene | 93 | 1508 | - | <0.01 | - | - |  |
| 74 | unknown 8 (sesqui) | 121 | 1512 | - | - | 0.02 | - |  |
| 75 | unknown 9 (sesqui) | 121 | 1518 | - | - | 0.07 | - |  |
| 76 | unknown 10 (sesqui) | 91 | 1519 | - | - | 0.07 | - |  |
| 77 | unknown 11 (sesqui) | 97 | 1599 | - | - | 0.01 | - |  |
| 78 | caryophyllene oxide | 79 | 1611 | - | - | 0.02 | - |  |
| 79 | unknown 12 | 148 | 1681 | - | - | 0.64 | - |  |
| 80 | nonadecane | 57 | 1900 | 0.16 | 0.27 | - | - |  |
| 81 | unknown 13 (sesqui) | 69 | 1915 | - | - | 0.02 | - |  |
|  | Sum number of compounds |  |  | 51 | 50 | 63 | 33 | 27 (=33.3% of the total 81) |

Table S2. Shannon diversity and richness of compounds in hexane surface washes of pollen of five chemotypes of *T. vulgare*. Displayed are parameter estimates and standard error values for the (generalised) linear mixed models ((G)LMM) and levels of significance for differences between chemotypes. Random factors in all models were maternal genotype (MG) and bloom onset (BO; i.e. the day on which the first mature flower was observed) and the number of groups for variance estimates is given in brackets. The total amount of produced pollen of each plant individual was included as fixed effect in models, to test factor interactions. Variance estimates for the random effects are shown for the full model, including the interaction, if the interaction was significant, otherwise the variance estimates refer to the minimised model, excluding the interaction.

| **Response factor, pollen data** | **Fixed effects** | **Parameter estimates ± s.e. for chemotypes** | | | | | **Variance estimates ± s.e. of random effects** | **Statistical test and result** |
| --- | --- | --- | --- | --- | --- | --- | --- | --- |
|  |  | *β*-thu. mono | art.ket mono | *α*, *β*-thu. mix | art. acet. mix | myrox. mix |  |  |
| **Shannon diversity** | chemotype, pollen amount | 1.02 ± 0.19  N = 9 | 1.05 ± 0.26  N = 7 | 1.04 ± 0.18  N = 6 | 1.2 ± 0.18  N = 9 | 1.12 ± 0.17  N = 7 | MG: 0.05 ± 0.23  (g = 11)  BO: 0.02 ± 0.13  (g = 19) | LMM (Poisson, log link)  CTxPA χ^2^ = 5.46, P = 0.38  CT χ^2^ =2.17, P = 0.71  PA χ^2^ = 0.74, P = 0.39 |
| **Compound richness** | chemotype, pollen amount | 16.11 ± 0.88  N = 9 | 15.29 ± 1  N = 7 | 14.71 ± 1  N = 6 | 14.33 ± 0.88  N = 9 | 15.67 ± 1.08  N = 7 | MG: 0.00 ± 0.00  (g = 11)  BO: 6.76 ± 2.6  (g = 19) | GLMM (Poisson, log link)  CTxPA χ^2^ = 1.1, P = 0.89  CT χ^2^ = 1.17, P = 0.86  PA χ^2^ = 0.01, P = 0.7 |

Table S3. Olfactometer assay testing the choice of florivorous beetles (*Olibrus* spp.) between premature (prior anthesis) and mature (pollen presenting) flower heads of five chemotypes of *T. vulgare*. Prior statistical analyses, the beetle duration of stay data was averaged across the four beetles tested per plant individual. Displayed are parameter estimates and standard error values of linear mixed models (LMM, Poisson, log link), comparing the percentage share of the duration of stay above olfactometer fields. Values and significance levels are given for the different chemotypes, which were tested in separate trials. The fixed effect in all models was the presented flower head (FH): premature, mature with pollen or none (control). Plant individual was included as random factor in all models and the number of groups (g) is given in brackets. Significant values (P < 0.05) are highlighted in bold and pointing up ▲ or down ▼ triangles highlight the highest and lowest parameter estimate, respectively.

| **Chemotype** | **Trials** | | **Parameter estimates ± s.e. for the duration of stay [%] above olfactometer fields** | | | **Variance estimates ± s.e. of random effect (plant)** | **LMM result** |
| --- | --- | --- | --- | --- | --- | --- | --- |
|  | Plants | Beetles | premature FH | mature FH | control (no FH), mean of both fields |  |  |
| *β*-thu. mono | 8 | 32 | 34.6 ± 2.5▲ | 16.3 ± 2.5▼ | 24.4 ± 2.2 | 5.2 ± 7.2 (g = 8) | χ^2^ = 389.4,  **P < 0.001** |
| art.ket mono | 11 | 44 | 21.3 ± 5.4 | 27.6 ± 5.4 | 25.2 ± 5.2 | 3.2 ± 17.9 (g = 11) | χ^2^ = 71.3,  P = 0.69 |
| *α*, *β*-thu. mix | 9 | 36 | 28.8 ± 2.7 | 19.3 ± 2.7 | 24.8 ± 2.6 | 5.2 ± 7.2 (g = 9) | χ^2^ = 283.6,  P = 0.065 |
| art. acet. mix | 8 | 32 | 29.1 ± 3.2▲ | 13.4 ± 3.2 ▼ | 27.6 ± 2.9 | 6.4 ± 8 (g = 8) | χ^2^ = 219.8,  **P = 0.002** |
| myrox. mix | 10 | 39 | 25.1 ± 3 | 23.8 ± 3 | 25.6 ± 3 | 9.8 ± 9.9 (g = 10) | χ^2^ = 227.4,  P = 0.89 |

Table S4. Florivore beetle abundance and abundance of mines on/in flower heads of four chemotypes of *T. vulgare*, planted in homogenous (one chemotype) and heterogenous (mixed chemotypes) plots in a semi-field experiment. Displayed are parameter estimates and standard error values for the generalised linear mixed models (GLMM) for differences in florivore abundance between chemotypes. The random factors in both models were plot nested in block. The number of groups for variance estimates is given in brackets. The plot type (PT, homogenous = individuals in one plot are of the same chemotype, heterogenous = individuals of different chemotypes are mixed in one plot) was included as fixed effect in models for beetle abundance, to test factor interactions. Mines were only counted in homogenous plots.

| **Response factor** | **Fixed effects** | **Parameter estimates ± s.e. for chemotypes** | | | | **Variance estimates ± s.e. of random effects** | **Statistical test and result** |
| --- | --- | --- | --- | --- | --- | --- | --- |
|  |  | *β*-thu mono | (Z*)*-chrys. acet. mono | camphor | *(E)-*carvyl acet. mono |  |  |
| **florivorous *Olibrus* spp./ plant** | chemotype x plot type | 0.7 ± 0.2  N = 18 | 0.7 ± 0.2  N = 18 | 0.5 ± 0.2▼ N = 18 | 0.8 ± 0.2▲ N = 18 | plot:block =  0 ± 0.2 (g = 6)  block = 0 ± 0.1 (g = 3) | GLMM  (Poisson, log link)  CTxPT χ^2^ = 13.1, **P = 0.002** |
| **mines/ plant** | chemotype | 3.1 ± 0.5  N = 24 | 3.8 ± 0.5▲  N = 24 | 1.4 ± 0.5▼  N = 24 | 3.3 ± 0.5  N = 24 | plot:block =  0 ± 0.2 (g = 12)  block = 0 ± 0.1 (g = 3) | GLMM  (Poisson, log link)  CT χ^2^ = 11.35, **P = 0.025** |
